# Studying dynamic stress effects on the behaviour of THP-1 cells by microfluidic channels

**DOI:** 10.1038/s41598-021-93935-w

**Published:** 2021-07-13

**Authors:** Semra Zuhal Birol, Rana Fucucuoglu, Sertac Cadirci, Ayca Sayi-Yazgan, Levent Trabzon

**Affiliations:** 1 Department of Nanoscience and Nanoengineering, Istanbul Technical University, 34469 Istanbul, Turkey; 2 Department of Molecular Biology and Genetics, Istanbul Technical University, 34469 Istanbul, Turkey; 3 Department of Mechanical Engineering, Istanbul Technical University, 34437 Istanbul, Turkey; 4 Nanotechnology Research and Application Center-ITUnano, Istanbul Technical University, 34469 Istanbul, Turkey; 5 MEMS Research Center, Istanbul Technical University, 34396 Istanbul, Turkey

**Keywords:** Biological techniques, Biotechnology, Cell biology, Molecular biology, Biomarkers, Cardiology, Diseases, Engineering, Nanoscience and technology

## Abstract

Atherosclerosis is a long-term disease process of the vascular system that is characterized by the formation of atherosclerotic plaques, which are inflammatory regions on medium and large-sized arteries. There are many factors contributing to plaque formation, such as changes in shear stress levels, rupture of endothelial cells, accumulation of lipids, and recruitment of leukocytes. Shear stress is one of the main factors that regulates the homeostasis of the circulatory system; therefore, sudden and chronic changes in shear stress may cause severe pathological conditions. In this study, microfluidic channels with cavitations were designed to mimic the shape of the atherosclerotic blood vessel, where the shear stress and pressure difference depend on design of the microchannels. Changes in the inflammatory-related molecules ICAM-1 and IL-8 were investigated in THP-1 cells in response to applied shear stresses in an continuous cycling system through microfluidic channels with periodic cavitations. ICAM-1 mRNA expression and IL-8 release were analyzed by qRT-PCR and ELISA, respectively. Additionally, the adhesion behavior of sheared THP-1 cells to endothelial cells was examined by fluorescence microscopy. The results showed that 15 Pa shear stress significantly increases expression of ICAM-1 gene and IL-8 release in THP-1 cells, whereas it decreases the adhesion between THP-1 cells and endothelial cells.

## Introduction

In the cardiovascular system, vessel functions are controlled by a combination of various factors such as mechanical forces, biochemical signals, nutrition, drugs, and interactions between different cell types that exist in circulation. The pathological conditions of the blood vessel, such as atherosclerosis, are dependent on the balance between these factors^1^. Wall shear stress is one of the mechanical forces, which is defined as the force per unit area that acts on a surface of endothelium lining the vessel wall^2^. In vivo, the level of shear stress in veins is low in magnitude at 0.076–0.76 Pa, due to the non-pulsatile character of the venous system. On the other hand, the levels of shear stress in large arteries and arterioles are in the range of 1–7 Pa and 2–6 Pa respectively, due to the laminar and pulsatile nature of flow in the arterial side of the circulation^3–8^.

Since the endothelial cells are in direct contact with blood flow, the shape and alignment of these cells are modified by changes in the type of flow and level of shear stress. When the blood flow is steady and unidirectional, endothelial cells are spindle-shaped and aligned with their long axis in the direction of flow, whereas their shape is more polygonal with a non-uniform orientation when the flow is turbulent or stagnant. The mechanism underlying this morphological adaptation is the cytoskeletal reorganization driven by actin filaments in stress fibers in endothelial cells^9–11^. It can be possible that the same mechanism can be active in monocyte cells exposed to different shear stresses. In this respect, the cytoskeletal organization and adhesion molecules have been investigated in this study since there is not clear information in the literature about the status of monocytes under different shear stress conditions.

Studying the mechanical forces on the cells needs a multi-disciplinary approach consisting of medicine, cell biology, molecular biology, physics, and engineering so that the problem can be investigated in a holistic way. Besides molecular approaches, multi-scale engineering techniques can help a better understanding of complex cellular functions^12^. Microfluidic platforms are very attractive for studying the effects of mechanical forces on cells, since the design of microfluidic systems makes it possible to deal with variations in shear stress in microchannels. In particular, microfluidic channels are able to be used with minimal quantities of samples and reagents, high resolution image analysis, low cost and portability^13^. Microfluidics enables the perfusion of long-term cell cultures by providing conditioned fluid flow and shear stress, and for these reasons it is used frequently in vascular modeling^14^. Chau et al. fabricated a microfluidic shear device that simultaneously evaluates ten different shear stresses on HUVEC cells (Human Umbilical Vein Endothelial Cells), unlike parallel-plate flow chambers^15^. Shao et al. designed an integrated microfluidic platform that enables assessment of pulsatile and oscillatory shear stress on endothelial cells^16^. Schimek et al. developed a chip-based system consisting of microfluidic channels to mimic the transport function of the human cardiovascular system using HDMEC cells (Human Dermal Microvascular Endothelial Cells)^17^.

This study focused on the response of THP-1 cells that were subjected to shear stress in the physiological range (4 Pa) and above (15 and 45 Pa) for 0.5 h and 3 h, in separate runs, under an continuous flow cycle by a syringe pump. Based on the Computational Fluid Dynamics (CFD) studies in this work, we examine THP-1 cells exposed to varying shear stress depending on the dimension of the cavities through the microchannel. As it is well known that the time varying stresses would be more damaging and effective on possible change of materials properties compared to the level of static stresses applied on^18^. Therefore, early failure and loss of performance of materials in fatigue conditions are to be expected when using a regime of time-varying stresses. The same problem may occur in the biological systems in which cells are exposed to time-varying stresses, and this is very similar to material fatigue, which causes early failure in the body. Therefore, we hypothesized that continuous change in shear stress and pressure may cause THP-1 cell fatigue since the cells need to change their morphology while they pass through microchannels with changing shear stress and pressure in each cavity. The shear stress-dependent immune molecules intercellular adhesion molecule-1 (ICAM-1) and interleukin-8 (IL-8) were investigated to assess any change in them as a result of time-varying stress changes. According to the literature, ICAM-1 mRNA expression and IL-8 release in HUVEC cells were changed by application of shear stress^19^; thus, here we decided to assess these molecules in monocytes subjected to various different shear stress conditions. In addition, adhesion between ECs and THP-1 cells was examined after exposing monocytes alone to shear stress. Results showed that 15 Pa shear stress increased the synthesis of ICAM-1 mRNA expression and IL-8 release, whereas there was a decrease in the number of THP-1 cells adherent to endothelial cells.

## Materials and methods

### Microchannels

#### Design

**Figure 1:**
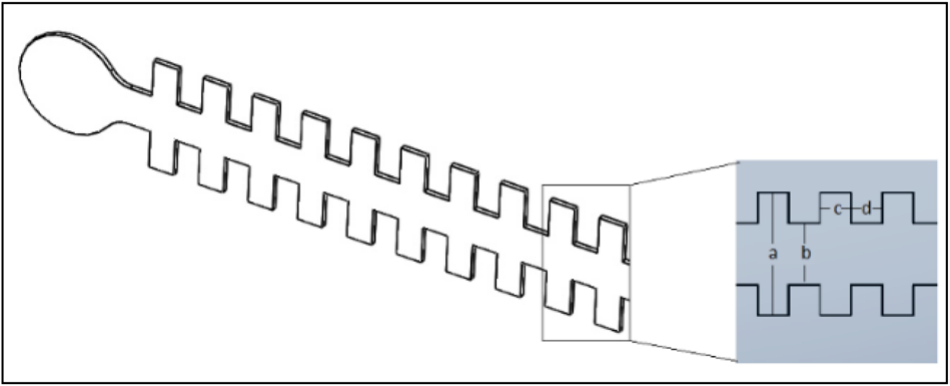
The 3D design of microchannels with cavities. Aspect ratio (a/b) was defined as three (3) for all configurations with various dimensions. The values of the parameters c and d are equal.

Two microchannels with different dimensions have been used in the experiments for creating different wall shear stresses on the bottom walls and pressure levels in the mid-section of the flow fields. Microchannel-A has been used to create 4 Pa under 2400 μl/min fluid flow and microchannel-B was used to create 15 Pa under 1200 μl/min fluid flow and 45 Pa under 3600 μl/min fluid flow by syringe pump. The 3D model of both microchannels is represented in Fig. 1.

The height of microchannels is 100 µm. The dimensions of the microchannels are shown in Table 1.

**Table 1:** The dimension of the cavitated microchannels.

Channel	a (µm)	b (µm)	c (µm)	d (µm)
A	2250	750	500	500
B	500	1500	1000	1000

#### Computational fluid dynamics analysis

The meshes for the CFD analysis have been generated by ANSYS-Meshing. After intensive mesh-analysis tests mesh-independent solutions have been obtained for the pressure difference between the inlet and outlet of the microchannel and for the wall shear stress thus, appropriate mesh resolution is determined for numerically-accurate solutions.

##### Boundary conditions and solution method

The CFD analysis is performed by ANSYS-Fluent where the flow is incompressible, steady and laminar. Working fluid is water with a constant density ρ of 1000 kg/m^3^ and dynamic viscosity µ of 10^–3^ kg/ms. The boundary conditions are as follows: uniform velocity is imposed at the channel inlet, pressure-outlet is defined at the channel exit and all walls have no-slip boundary condition. In this regard, continuity and Navier–Stokes equations have been solved iteratively by finite-volume-based flow solver using SIMPLE algorithm until the convergence criteria are achieved.

##### Theoretical wall shear stress calculation

Wall shear stress is defined as the tangential stress parallel to the surface caused by the blood flow that affects endothelial cells lining on blood vessels by direct action with the flow or indirect action with transporting chemicals and similar compounds. Reynolds number (Re), defined as the ratio of the inertial forces to the viscous forces is calculated in the Eq. (1):

1 $$Re=\frac{\rho u{D}_{h}}{\mu }$$

where μ is dynamic viscosity of the fluid, ρ is fluid’s density, D_h_ is the hydraulic diameter, and u is the bulk velocity of the liquid. Hydraulic diameter is the characteristic length, which depends on the geometry of the microchannel. In the current study, rectangular microchannels with cavities are used in the experiments and CFD analysis thus, the hydraulic diameter should be defined for both the wide section width w_i_ = a and the narrow section width w_i_ = b as given is the Eq. (2):

2 $${D}_{h}=\frac{4A}{P}=\frac{{2w}_{i}h}{{w}_{i}+h}$$

where A is the cross-sectional area and P is a wetted perimeter of the channel. The velocity profile for a fully developed laminar flow through a rectangular channel with periodically varying cross-section can be written as shown in Eq. (3), where U_i_ is the maximum velocity at either wide or narrow section of the microchannel, and h is the channel’s height.

3 $$u\left(y\right)=4{U}_{i}\left[\left(\frac{y}{h}\right)-{\left(\frac{y}{h}\right)}^{2}\right]$$

The parabolic function of the velocity satisfies no-slip boundary conditions at the lower and upper walls of the microchannel (y = 0 and y = h) and maximum velocity at the centerline (y = h/2). Hence, the volumetric flow rate can be calculated based on the bulk velocity which corresponds to two thirds of the centerline velocity for the laminar flow in rectangular channels as indicated in Eq. (4).

4 $$Q={w}_{i}\underset{y=0}{\overset{y=h}{\int }}u\left(y\right)dy=\frac{2}{3}{U}_{i}{w}_{i}h$$

For Newtonian-type fluids, there is a linear relation between the shear stress and the rate of deformation. Equation (5) which yields a relation between the wall shear stress and the maximum velocity depending on the contraction or expansion side of the channel can be derived from Eq. (3).

5 $${\tau }_{wall}={\left.\mu \frac{du}{dy}\right|}_{y=0,h}=\pm \frac{4\mu {U}_{i}}{h}$$

Shear stress is a biomechanical force that can be calculated using fluid dynamics approaches. Hagen–Poiseuille equation in vessel structure with constant circular diameter gives a parabolic velocity profile for laminar and steady blood flow and shear stress can be obtained which is directly proportional to blood flow rate and inversely proportional to diameter^3,20–24^. In a similar way, wall shear stress calculation can be adopted to the rectangular channel with varying cross-section. Equation (6) can be obtained by substituting the maximum velocity in terms of the volumetric flow rate from Eq. (4) and provides a general theoretical expression for the amplitude of wall shear stress variation at either expansion or contraction of the microchannel.

6 $${\tau }_{wall}=\pm \frac{6\mu Q}{{w}_{i}{h}^{2}}$$

As given in the theoretical calculation, the CFD analysis revealed a periodically changing wall shear stress along the mid-line on the bottom surface of the microchannel assisting an understanding of the effects of the biomechanical forces on THP-1 cells.

#### Fabrication

Microchannels were formed by bonding one layer of a glass slide with one layer of PDMS (Sylgard 184; Dow Corning, Midland, USA) and the microfluidic channels were fabricated by soft lithography. 100 μm thick SU-8 3050 photoresist (MicroChem Corp., Newton, USA) was deposited on Si-wafer (University Wafer, Boston, USA) and distributed by a spinner (Laurell WS 400 B, North Wales, USA). Then SU-8 was processed to be patterned by mask aligner (OAI Model 200, San Jose, USA) under UV light. The PDMS was mixed in a 10:1 ratio with the curing agent and poured on the SU-8 master after removing bubbles under vacuum. Then, the cast was cured at 80 °C for 2 h and incubated in the oven overnight to obtain a PDMS replica of microchannel features. Following day, the PDMS mold was removed from the SU-8 master, and then input and output holes were drilled to create tubing connection by using a 0.5 mm OD biopsy puncher (Elveflow, Paris, France). Finally, PDMS and glass slides were exposed to medium Radiofrequency (RF) power for 1 min to provide surface modification for bonding in the plasma device (Harrick Plasma device (New York, USA)^25^.

### Cell culture

#### THP-1 cell culture

THP-1(human monocytic) cells were kindly provided by Prof. Nesrin Ozoren (Bogazici University, Istanbul, Turkey) and maintained in a humidified cell culture incubator at the 37 °C temperature and 5% CO_2_ conditions in complete RPMI 1640 medium (Gibco, Thermo Fisher Scientific, Waltham, USA) supplemented with 10% fetal bovine serum (FBS) (Biowest, Nuaille, France) and 1% penicillin–streptomycin (Lonza, Basel, Switzerland) in the upright position. The confluency of cells was assessed daily by using a light microscope. The THP-1 cells were split at 80% cell confluency. The entire contents of the flask were removed and centrifuged at 125×*g* for 5 min. The pellet was resuspended in 10 ml complete RPMI-1640 medium and counted on the Thoma cell counting chamber by Trypan blue staining (1:10). Cell density was adjusted to 4 × 10^5^ viable cells in the complete medium^26^.

#### HUVEC cell culture

HUVEC cell line was purchased from Lonza (catalog number CC-2517) and maintained in a humidified cell culture incubator at the 37 °C temperature and 5% CO_2_ conditions in complete M199 medium (Gibco) supplemented with 10% fetal bovine serum (FBS) (Biowest) and 1% penicillin–streptomycin (Lonza). The confluency of cells was assessed daily by using a light microscope. The HUVEC cells were split at 80% cell confluency. The cells were washed with 3–5 ml 1× PBS (Biochrom, Cambridge, UK). The cells were trypsinized and split into a fresh T25 flask. containing M199 supplemented with 10% FBS and 1% Pen/Strep^27^.

### Shear stress application on THP-1 cells

All the microchannels and accessories were sterilized by UV exposure for 15 min before performing the experiments. After sterilization, microchannels were washed with complete RPMI-1640 medium to eliminate bubbles from the system. THP-1 cells were suspended in medium and were injected and circulated through the cavitated microchannels using the syringe pump system on continuous circulation under 4, 15, or 45 Pa shear stress, in separate, during 0.5 and 3 h. Experiments for THP-1 cells were performed with 10 ml cell suspension at 0.8 × 10^6^/ml cell density, and in parallel cells were incubated in a static environment (in cell culture flasks) at the same density throughout the experiment as a control^25^.

### Quantitative real time-PCR

Total RNA was isolated from THP-1 cells with the RNeasy Micro Kit (Qiagen, Hilden, Germany) according to the manufacturer’s instructions and quantified using a Nanodrop spectrophotometer. The corresponding cDNA was amplified using PCR, the High Capacity cDNA Synthesis Kit (Applied Biosystems, Thermo Fisher Scientific, Waltham, USA). Real-time RT-PCR reactions was performed using the SYBR Green PCR Master mix (Luna Universal Probe qPCR kit, New England Biolabs, USA) on ABI StepOne Real Time System. Expression level of ICAM-1 was normalized to 18S rRNA housekeeping gene and calculated using ΔΔCt method in three replicates. Primers sequences are as follows: ICAM-1 forward 5′-GCTTCGTGTCCTGTATGGC-3′, reverse 5′-AGTGGGAAAGTGCCATCCTTT-3′; 18s forward GGCCCTGTAATTGGAATGAGTC, reverse CCAAGATCCAACTACGAGCTT.

### Cytokine assay

Supernatants of each group were collected after THP-1 cells were subjected to 4, 15, and 45 Pa shear stress during 0.5 and 3 h. The IL-8 protein level in supernatants was analyzed using the IL-8 ELISAMAX kit (BioLegend, San Diego, USA) according to the manufacturer’s protocol. Absorbance readings were taken in Bio-Rad microplate reader at λ = 450 nm and 650 nm, respectively.

### Adhesion assay

Before the adhesion assay, HUVEC cells, which were seeded in a 6-well plate with 4 × 10^5^ cells/ml density, were grown to 80% confluence and later were pretreated with 10 ng/ml TNF-α for 4 h. After, THP-1 cells were stained with Hoechst 33342 (Thermo Fischer Scientific, Waltham, USA) (dilution 1:250 in RPMI 1640 per 1 × 10^6^ cells) at RT for 20 min. Stained THP-1 cells were washed with PBS for two times and resuspended in RPMI-1640 complete medium. After that, THP-1 cells were subjected to 15 Pa shear stress through cavitated microchannel-B under 15 Pa by ExiGo pump (Cellix Ltd., Dublin, Ireland) at refilling mode for 0.5 and 3 h in separate experiments. THP-1 cells in the static control group were not subjected to fluid flow. At the end of the experiment, three groups were placed in different falcon tubes in mixed medium (RPMI 1640-M199 in equal volume) and then added to HUVEC cells, which were seeded in the 6-well plates. After 1 h incubation, cells were washed with PBS for 3–4 times to remove unbound THP-1 cells on HUVEC cells. The number of adherent THP-1 cells were examined under Zeiss Axio A1 fluorescence microscope (Carl Zeiss Meditec, Jena, Germany) by counting five random fields in each group.

### Statistical analysis

GraphPad Prism 6.0 software was used for calculating all *p* values. ANOVA and Student’s *t* test were used to determine the *p* values according to existed number of data. The standard error of the mean was indicated with vertical bars in column graphs. *p* values of less than 0.05 were considered statistically significant.

## Results

### Flow rate and shear stress relation

**Figure 2:**
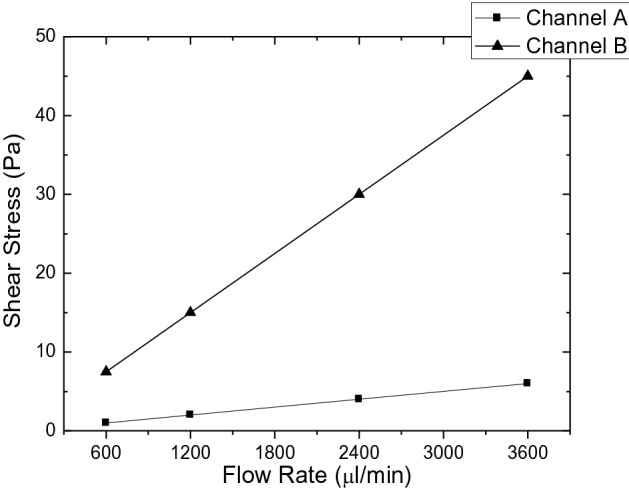
The comparative flow rate-shear stress graphic for microchannel-A and B.

The simulation studies were performed by CFD using ANSYS-Fluent software. The comparative flow rate-shear stress graphic for various flow rates in the cavitated microchannel-A and B is shown in Fig. 2.

**Figure 3:**
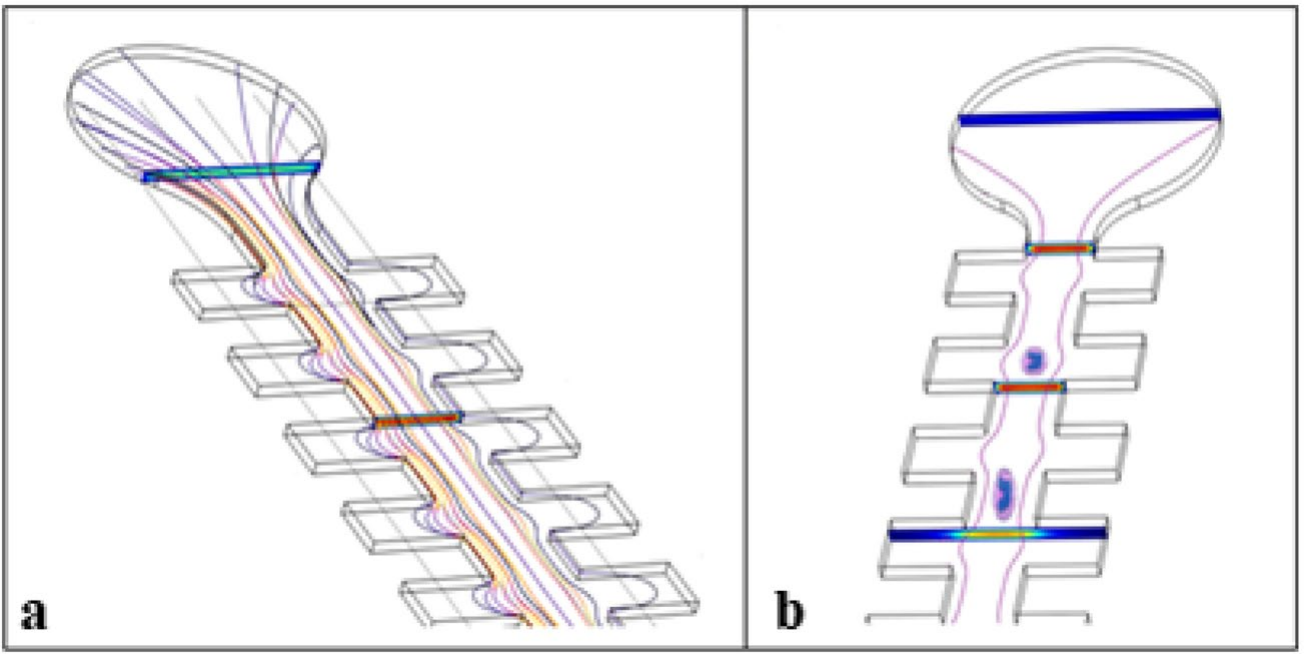
Schematic of the flow rate lines (**a**) and the morphology change of the THP-1 cells (**b**) inside the microchannel.

Channels A and B were chosen in the experiments to determine ICAM-1 mRNA expression and release of IL-8 from THP-1 cells, since the level of wall shear stress under test are in the range of target ones. The flow rates have been calculated by CFD to induce 4, 15, and 45 Pa of shear stress levels on THP-1 cells. 4 Pa is defined as in the range of physiological shear stress, whereas 15 and 45 Pa are above the physiological range. The difference between the minimum and maximum shear stress, called amplitude, that occurs through the microchannel is the shear stress acting on the cells. The flow rate lines are shown in Fig. 3a, and the morphology change of THP-1 cells passing through the cavities inside the microchannel, because of change in shear stress, is shown in Fig. 3b.

### 15 Pa significantly changes the shear stress relevant molecules

**Figure 4:**
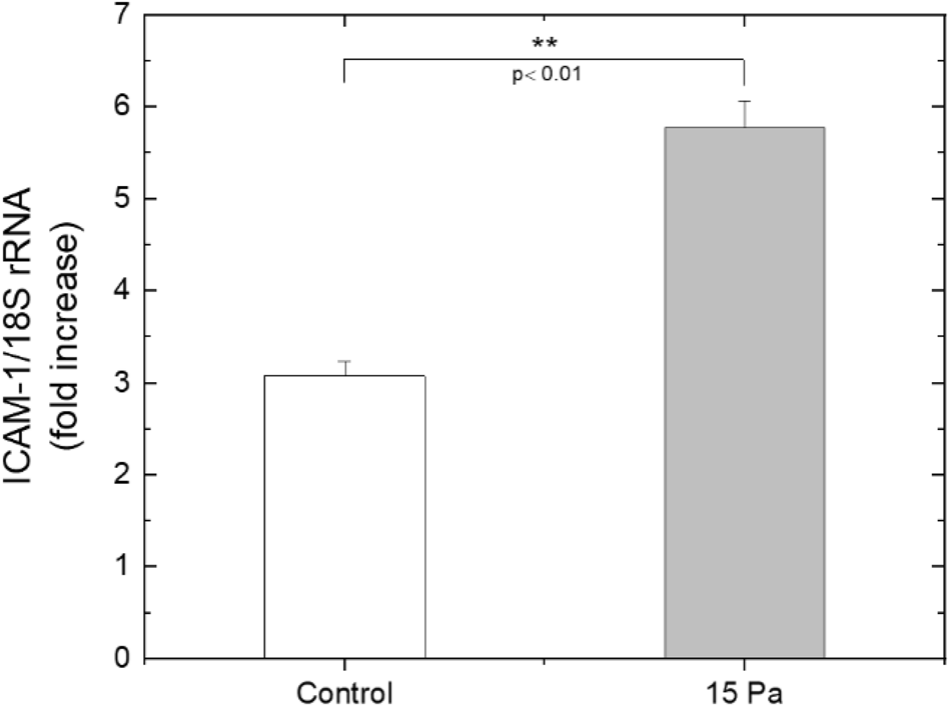
15 Pa of shear stress leads to an increase in ICAM-1 relative expression in THP-1 cells. THP-1 cells were either kept in a static environment (control) or treated with 15 Pa for 3 h (15 Pa). Bar graph indicates mean ICAM-1 expression levels analyzed by qRT-PCR from two independent experiments. Fold change expression of the ICAM-1 gene was normalized to18S rRNA. *p* values were determined by Student’s *t* test.

ICAM-1 has been investigated widely in endothelial cells subjected to various types of shear stress^19^ however, no detailed information has been published regarding any change in ICAM-1 mRNA levels in THP-1 cells after application of shear stress. Therefore, ICAM-1 mRNA expression levels in sheared THP-1 cells were determined by qRT-PCR. Microchannel-B was used to apply shear stress on THP-1 cells. THP-1 cells were subjected to laminar shear stress at 15 Pa for 3 h to assess the effect since the increase in the content of F-actin was determined to be highest after the treatment of 15 Pa, compared to the control and the other experimental groups in our previously published article^25^. As shown in Fig. 4, ICAM-1 mRNA levels in THP-1 cells treated with 15 Pa for 3 h were significantly higher than the control group.

**Figure 5:**
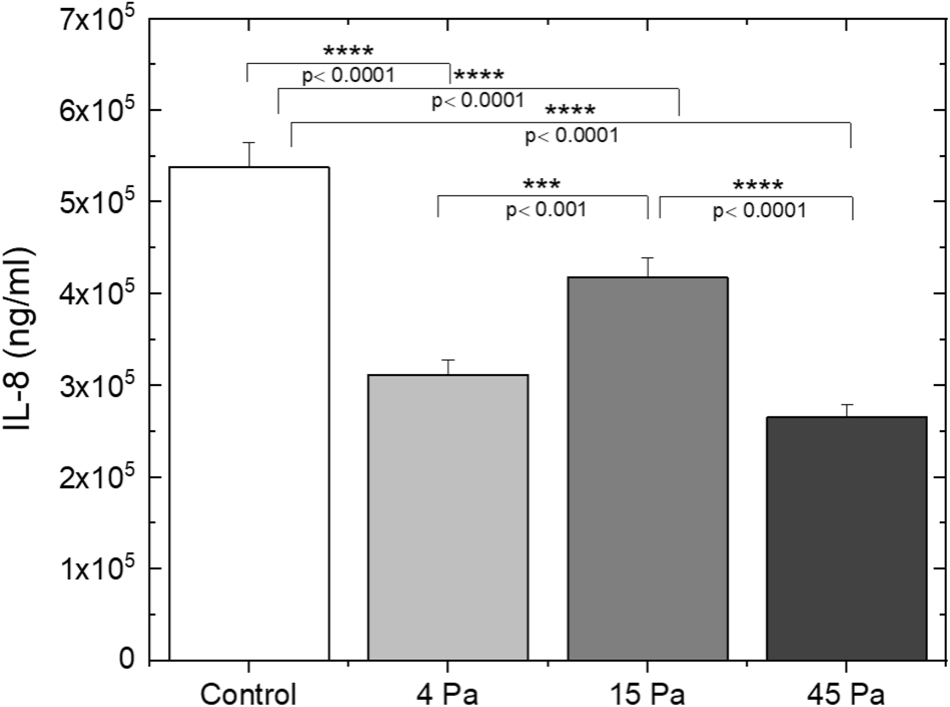
Various shear stress forces downregulate IL-8 secretion from THP-1 cells. THP-1 cells were treated with 4 Pa, 15 Pa, or 45 Pa shear stress for 3 h. IL-8 levels were measured by ELISA. Bar graph represents the mean IL-8 concentration of four independent experiments. *p* values determined by ANOVA (± SEM).

Next, we evaluated the effect of shear stress on IL-8 secretion from THP-1 cells. In this experimental setting, microchannel-A was used to create 4 Pa, and microchannel-B was used to develop 15 Pa and 45 Pa shear stress. As shown in Fig. 5, there was a significant decrease in IL-8 secretion from THP-1 cells, which were exposed to various shear stress forces (4 Pa, 15 Pa or 45 Pa) compared to the untreated control group. Although IL-8 secretion was reduced to its lowest level in THP-1 cells exposed to 45 Pa of shear stress, exposure to 4 Pa shear stress also led to a decrease to a closer value in these cells. Interestingly, 15 Pa shear stress did not lead to such a large reduction in IL-8 secretion in THP-1 cells compared to 4 Pa and 45 Pa treatments. Overall, these findings indicate that shear stress upregulated ICAM-1 expression, but downregulated secretion of IL-8 in THP-1 monocytic cells.

### Adhesion decreased between endothelial and THP-1 cells

**Figure 6:**
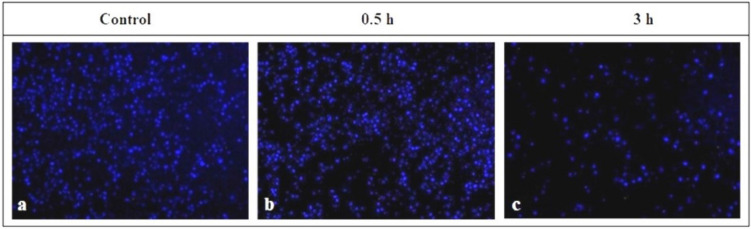
Pre-exposure of THP-1 cells to 15 Pa shear stress decreases their adhesion to HUVEC cells. THP-1 cells, which were pre-labeled with Hoechst 33342 (blue), were exposed to 15 Pa for 0.5 h or 3 h. THP-1 cells were then placed on TNF-alpha-pre-stimulated HUVEC cells for 3 h. After non-adherent THP-1 cells were removed, Hoechst 33342-stained-THP-1 cells (blue), which were attached to HUVEC cells, were monitored by a Zeiss Axio A1 fluorescence microscope. (**a**) THP-1 cells, which were placed on HUVEC cells in a static environment without exposing to a flow; (**b**) THP-1 cells, which were pre-exposed to 15 Pa shear stress for 0.5 h were placed on HUVEC cells, and (**c**) THP-1 cells, which were pre-exposed to 15 Pa shear stress for 3 h were placed on HUVEC cells. The magnification is 10×.

Having shown that shear stress in cavitated microchannels leads to changes in THP-1 cell behavior, we wondered whether attachment of THP-1 to endothelial cells (HUVEC) would be affected. To address this point, THP-1 cells, which were pre-labeled with Hoechst 33342, were subjected to 15 Pa shear stress with a continuous flow cycle for 0.5 h and 3 h periods. Later, THP-1 cells were placed on HUVEC cells, which were pre-stimulated with TNF-alpha to induce a pro-inflammatory environment. After removal of non-adherent THP-1 cells, the attachment of THP-1 to HUVEC cells was assessed using a fluorescence microscope and data are represented in Fig. 6.

**Figure 7:**
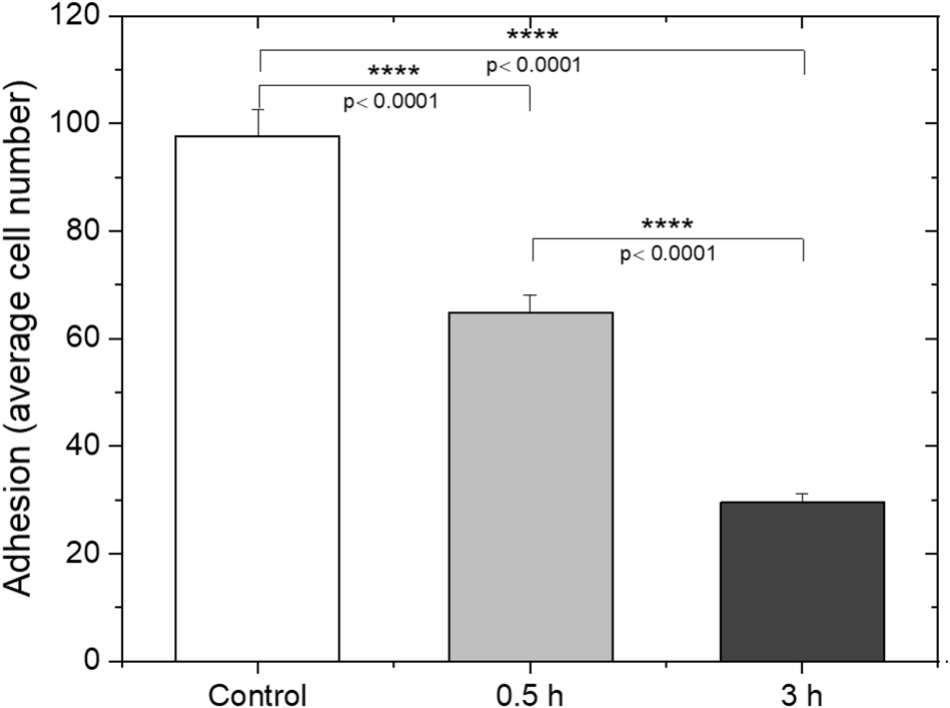
Shear stress attenuates the adhesion of THP-1 cells and HUVEC cells significantly. HUVEC cells were kept under static conditions, and THP-1 cells were subjected to shear stress at 15 Pa for 0.5 h and 3 h, separately, and later placed on HUVEC cells. THP-1 cells were stained with Hoescht 33342 cell tracker dye. 5 random fields were assessed to semi-quantify the adhesion level. Bar graph represents the mean value of adhesion based on two independent experiments. *p* values determined by ANOVA (± SEM).

As shown in Fig. 7, the THP-1 cell number attached to HUVEC cells decreased in groups exposed to shear stress for either 0.5 h and 3 h, compared to the untreated control group. Moreover, 3 h exposure to shear stress significantly attenuated the binding of THP-1 cells to HUVEC cells compared to both 0.5 h and control groups. This finding suggests that adhesion level to HUVEC cells decreases when the exposure time of shear stress at 15 Pa increases for THP-1 cells.

## Discussion

Mechanical forces take the first place to understand how cells and tissues emerge and develop an adaptation in the direction of changes of environment^28^. Studying the effects of the mechanical forces on cells in vivo is difficult, so there need to be alternative approaches and tools for their study in vitro^6^. Many of these techniques have different principles in operation, analysis, and detection. The microfluidic system is one of these techniques, which has the benefits of low wastage of reagents and samples, design flexibility, and control of the cellular environment^29^. We used cavitated microfluidic channels as a tool for investigating shear stress in the physiological and above range on THP-1 cells, to find out whether their response is similar to endothelial cells lining blood vessels. In addition, we determined whether the application of various shear stresses to cells, to induce fatigue, over various time periods, could induce a change in molecular expression in THP-1 cells, thereby reflected a functional change in these cells.

Vascular endothelial cells lining blood vessels are subjected to mechanical forces such as fluid shear circumferential distention, stress, and blood pressure; these play essential roles in the regulation of function and remodeling. Therefore, any abnormalities of mechanical forces can cause diseases such as atherosclerosis or hyperplasia^30^. The pathophysiological shear stress and disturbed flow in the blood vessels, sensed by endothelial cells, may directly act on the smooth muscle cells after endothelial damage^31^. This situation causes the release of proinflammatory molecules that make endothelial cells more adhesive to platelets and these endothelial-bound platelets may recruit leukocytes to the damage sites. This pathological interaction between platelets, endothelial cells, and leukocytes catalyzes the inflammatory process required for the development of atherosclerosis^32^. Based on observations from animal and human studies, the formation of plaque is intimately connected to hemodynamic factors. Cheng et al. developed a perivascular shear stress modifier that mimics regions which have different shear stress compositions (increased, lowered and lowered/oscillatory)^33^. They placed a cast around the vessel wall to create a fixed geometry, which causes gradual stenosis. As a result, in the vessel segment inside the cast, different regions include lowered shear stress, increased shear stress, and oscillatory shear stress with vortices occurring respectively by increasing or decreasing the blood flow. In our study, we considered a similar principle to create shear stress and pressure difference. The values of the shear stresses were evaluated as the difference (amplitude) between the wide and narrow regions in cavitated channels. These amplitude values have been used to calculate the flow rates by CFD using Ansys-Fluent software. The most important consideration when determining these flow rates was to perform experiments without damaging the cells seeded in the microchannels. This was why two microchannels were used in the experiments. In this content, the target shear stress values were determined as 4 Pa, which is within the physiological range, and 15 and 45 Pa which are above the physiological values, based on the literature^23^.

Although several studies have been performed on the F-actin network of endothelial cells, there is no clear information on how F-actin synthesis changing in THP-1 cells under shear stress. In our previous study^25^, we reported that the monocytic cells were subjected to 4, 15, and 45 Pa of shear stress for 0.5 and 3 h, to address this issue. The reason to choose 15 Pa target shear stress for investigating ICAM-1 mRNA is because this causes the greatest increase in rate of F-actin content at 15 Pa for 0.5 h, compared to the control group in our previous study. ICAM-1 is a leukocyte adhesion molecule that is expressed by endothelial cells in altered shear stress, inflammation, and pathophysiological conditions such as hyperplasia and atherosclerosis. Even though it is known that ICAM-1 is expressed in monocytes^34^, this has not been investigated under different shear stress levels. According to our results, the ICAM-1 mRNA level in THP-1 cells was found to be significantly higher than the control group under 15 Pa shear stress. The results obtained from the experiments of ICAM-1 expression in THP-1 cells, when exposed to shear stress, are promising in terms of being pioneering work in the role of monocytes in atherosclerotic plaque formation.

For neutrophils and lymphocytes, IL-8 is a chemotactic cytokine (chemokine) that is produced by many cells, including fibroblasts, neutrophils, monocytes, endothelial cells, and cancer cells^35^. IL-8 is known to be found in human atherosclerotic plaque regions. The cells which have fewer receptors for this chemokine are less sensitive to atherosclerosis, and also in lesions, fewer monocytes could be found. Gerszten et al. investigated how chemokines play a role in the interaction of monocytes subjected to flow (shear stress of 2 Pa) in the vascular endothelium^36^. They observed that IL-8 triggers the strong adhesion between monocytes and endothelium, which shows an unexpected role for IL-8 in monocyte recruitment, and this chemokine can rapidly cause rolling monocytes to adhere tightly to form monolayers. In this study, IL-8 release was investigated after shear stress application at 4, 15, and 45 Pa to determine the shear stress value that causes changes at maximum range in THP-1 cells. It was found that the IL-8 release in each group was decreased compared to control. Exposure of THP-1 cells to 4 Pa and 45 Pa shear stress leads to attenuation of IL-8 secretion, with higher levels in the latter. Interestingly, even though exposure of THP-1 cells to 15 Pa shear stress caused a decrease in IL-8 secretion, it was not as substantial as at the other shear forces. Although, IL-8 is an attractant with in vitro activity for neutrophils, lymphocytes, and basophils, it is not for monocytes^37^. It can be suggested that shear stress between 4 and 45 Pa can reduce the release of IL-8 from THP-1 cells compared to the control group, which is important to activate the neutrophil adhesion to the endothelium that causes the vascular environment to be less atherogenic.

Studies show that many molecules can act as sensory elements to transduce hydrodynamic forces applied to endothelial cells^38^. The adhesion of leukocytes to activated endothelium via high-level expression of adhesion molecules is influenced by low shear stress^39^. In the case of atherosclerotic plaque development, firstly, monocytes adhere to endothelial cells in the inflammatory site, and then endothelial transmigration follows the adhesion. The process starts with the initial capture step (tethering) and proceeds with rolling, firm adhesion, and migration, respectively. Different adhesion molecules expressed on the surface of activated endothelial cells play a role in each step^40^. The interaction between l-selectin on the monocytes and its ligand on the endothelial cells leads to a rolling motion after the first contact between monocyte and endothelial cells in the second step^41,42^. β1 integrin VLA-4 (CD49d) of monocytes interacts with vascular cell adhesion molecules 1 (VCAM-1; CD106) on endothelial cells in the third step. Finally, β2 integrin interacts with ICAM-1 (CD54), and transendothelial migration may occur in by means of several molecules such as ICAM-1, VCAM-1, and platelet endothelial cells adhesion molecule-1 (PECAM-1; CD31)^42–44^. In the literature, there is no clear information about the investigation of the adhesion characteristic after treating monocytes with shear stress (continuous cycling) for the long term and higher physiological levels. Hsiai et al. developed a microelectromechanical systems (MEMS) sensor to quantify individual monocyte/EC binding kinetics in terms of displacement and velocity profiles at ± 0.26 Pa shear stress under oscillatory flow. According to those results, the application of oscillatory flow, low mean shear stress, and high shear stress gradient up-regulated the expression of ICAM-1, in parallel with the increasing number of monocytes bound to EC^45^. However, in our study, although an increase in ICAM-1 gene expression was detected when THP-1 cells were subjected to shear stress at 15 Pa under laminar flow for 3 h compared to control, the adhesion of THP-1 cell to endothelial cells was decreased. This discrepancy is most likely caused by changes in different adhesion molecules, which may be more dominant in determining the adhesion behavior of monocytes to endothelial cells at higher shear stress levels.

## Conclusion

The microfluidic channels have been designed and fabricated to create and apply shear stress on endothelial and monocytes in terms of investigating their effects on these cells. Shear stress was applied by two microchannels, where different shear values were obtained through manipulating the geometry and dimension of the microchannels. The shear stress levels used in the experiments were determined with simulation studies by the ANSYS-Fluent program. These microchannels were designed to create calculated shear stress as well as mimicking the atherosclerotic environment by the use of cavities.

The activation of endothelial cells by up-regulation of cell adhesion molecules and chemokines causes monocyte cell recruitment; thus, the accumulation of monocytes in the wall of arteries plays a major role in the process of atherosclerosis^46^. Since any change in shear stress level affects directly these cells by the blood flow, which may be physiological or pathological such as in atherosclerosis, endothelial cells have been widely investigated in terms of their changes in structural and adhesion molecules in response to stress. However, no information is available regarding the status of these molecules in monocytes in response to shear stress. Therefore, ICAM-1 mRNA expression levels were investigated to find out whether or not shear stress affects the same molecules in monocyte cells as in endothelial cells. In addition, chemokines play a significant role in the communication between endothelial and monocyte cells in atherosclerosis^47^. Various cell types, including activated endothelial cells, release IL-8 for the recruitment of monocytes, and this chemokine is found in human atherosclerotic plaques^48^. IL-8 release was investigated here in monocytes activated by flow for various periods to find out how the change in shear stress level affects the level of IL-8 release. In the case of atherosclerotic plaque development, firstly monocytes adhere to endothelial cells at the inflammatory site, and then endothelial transmigration follows the adhesion. The adhesion of THP-1 cells to endothelial cells was investigated to determine the adhesion characteristics after treating THP-1 cells with shear stress.

This shear stress study aimed to fill some gaps that exist in our understanding of monocyte biology, which contributes to inflammation in atherosclerotic plaques. The results show that treating THP-1 cells with 4, 15, and 45 Pa shear stress induces significant changes in shear stress-related molecules such as ICAM-1 and IL-8 and also determines the behavior of THP-1 cells in adhesion to the endothelium as well. The results showed that 15 Pa triggers significant changes in the shear stress-related molecules. Moreover, in the condition of continuous cycling of monocytes through cavitated microchannels, cells are exposed to periodic and stable average higher and lower shear stress levels; thus, this condition can cause cell fatigue.

## Data Availability

The data that support the findings of this study are available from the corresponding author, [SZB], upon reasonable request.
